# Application of Spatial Analysis on Electronic Health Records to Characterize Patient Phenotypes: Systematic Review

**DOI:** 10.2196/56343

**Published:** 2024-10-15

**Authors:** Abolfazl Mollalo, Bashir Hamidi, Leslie A Lenert, Alexander V Alekseyenko

**Affiliations:** 1 Biomedical Informatics Center Department of Public Health Sciences Medical University of South Carolina Charleston, SC United States

**Keywords:** clinical phenotypes, electronic health records, geocoding, geographic information systems, patient phenotypes, spatial analysis

## Abstract

**Background:**

Electronic health records (EHRs) commonly contain patient addresses that provide valuable data for geocoding and spatial analysis, enabling more comprehensive descriptions of individual patients for clinical purposes. Despite the widespread use of EHRs in clinical decision support and interventions, no systematic review has examined the extent to which spatial analysis is used to characterize patient phenotypes.

**Objective:**

This study reviews advanced spatial analyses that used individual-level health data from EHRs within the United States to characterize patient phenotypes.

**Methods:**

We systematically evaluated English-language, peer-reviewed studies from the PubMed/MEDLINE, Scopus, Web of Science, and Google Scholar databases from inception to August 20, 2023, without imposing constraints on study design or specific health domains.

**Results:**

A substantial proportion of studies (>85%) were limited to geocoding or basic mapping without implementing advanced spatial statistical analysis, leaving only 49 studies that met the eligibility criteria. These studies used diverse spatial methods, with a predominant focus on clustering techniques, while spatiotemporal analysis (frequentist and Bayesian) and modeling were less common. A noteworthy surge (n=42, 86%) in publications was observed after 2017. The publications investigated a variety of adult and pediatric clinical areas, including infectious disease, endocrinology, and cardiology, using phenotypes defined over a range of data domains such as demographics, diagnoses, and visits. The primary health outcomes investigated were asthma, hypertension, and diabetes. Notably, patient phenotypes involving genomics, imaging, and notes were limited.

**Conclusions:**

This review underscores the growing interest in spatial analysis of EHR-derived data and highlights knowledge gaps in clinical health, phenotype domains, and spatial methodologies. We suggest that future research should focus on addressing these gaps and harnessing spatial analysis to enhance individual patient contexts and clinical decision support.

## Introduction

Electronic health records (EHRs) have significantly enriched clinical decision support by providing relatively cost-effective, time-efficient, and convenient sources of a large population of patient records [1,2]. Because EHRs often contain patient addresses, spatial analysis can enable value addition via high-resolution geocoding. The simplest of such analyses may be mapping, which can promote a better understanding of health disparities. Further, patient geocoding can link external data such as environmental, demographic, and socioeconomic factors for more refined patient phenotyping and a more profound understanding of patient exposures for targeted interventions [3].

The possibilities for applying spatial analysis on individual-level, EHR-derived data are beyond geocoding, basic mapping, or external data linkage. For instance, spatial network analysis examines proximity to the sources of pollution [4], measures accessibility to health care facilities [5], and optimizes resource allocations to mitigate health disparities [6]. Spatial clustering pinpoints statistically significant spatial and spatiotemporal hotspots and cold spots [7], especially when considering longitudinal EHRs data. Moreover, spatial and spatiotemporal modeling can identify localized patterns, trends, and relationships within a specific region [8,9]. Identifying underserved communities through spatial analysis can enhance clinical decision support to implement targeted interventions such as screening, vaccination, or health education campaigns.

Despite the availability of advanced spatial analysis methods, most studies primarily focus on basic mapping or geocoding. Moreover, while these methodologies have the potential to better describe the context of individual patients in biomedical studies, there is a need for their improved application to derive more meaningful insights. To accurately address medical conditions, identify a disease in a patient, and scale that to cohorts of patients, phenotyping is required [10]. Phenotypes are a combination of observable traits, symptoms, and characteristics. They can contain inclusion and exclusion criteria (eg, diagnoses, procedures, laboratory reports, and medications) and can be used to recruit patients who fit the necessary criteria for clinical trials.

A prior systematic review used spatially linked EHRs data to investigate the effects of social, physical, and built environments on health outcomes [11]. Another study highlighted the need to integrate spatial data related to individual patients into health care decision-making and practice [12]. Nonetheless, this is the first comprehensive study that systematically reviews US-based studies that used spatial analysis for analyzing EHR-derived data in characterizing patient phenotypes for clinical decision support and interventions. This review collates and synthesizes existing literature that used individual-level health data from EHRs in conjunction with advanced spatial analyses and patient phenotyping. Thus, the main objectives of this review are (1) to evaluate the degree to which advanced spatial methods are currently being used with individual-level data sourced from EHRs in the United States, (2) to identify areas of spatial analyses most applicable to biomedical studies, (3) to categorize publications concerning their biomedical and clinical areas and the specific patient phenotypes they target, and (4) to highlight knowledge gaps and propose future research directions for harnessing the potential of spatial analysis to enhance the context of individual-level data sourced from EHRs for biomedical studies.

## Methods

### Overview

This systematic review was performed using the protocols outlined by the PRISMA (Preferred Reporting Items for Systematic Reviews and Meta-Analyses) to identify the studies that satisfy the eligibility criteria for subsequent data extraction and synthesis (Multimedia Appendix 1).

### Data Source

A comprehensive search for peer-reviewed studies was carried out using abstracts and titles screening within the PubMed/MEDLINE, Scopus, and Web of Science databases using the search terms in Table 1. The search was conducted on August 29, 2023, without limitations on study design or specific health domains.

**Table 1:** The search strategy key terms.

Theme^a^	Key terms
Spatial analysis	(“Geospatial*” OR “Geo-spatial*” OR “Spatio-Temporal” OR “Spatial Temporal” OR “Space-Time” OR “Space Time” OR “Spatiotemporal” OR “Geocod*” OR “ Spatial Autocorrelation” OR “Spatial Interpolation” OR “Spatial Epidemiology” OR “Spatial Data” OR “Spatial Modeling” OR “Spatial Modelling” OR “Spatial Mapping” OR “Geographic Mapping” OR “Georeferenc*” OR “Spatial Analys*” OR “Spatial Inequalit*” OR “Spatial Disparit*” OR “Spatial Dependenc*” OR “Spatial Access*” OR “Geographical Mapping” OR “Geographical Visualization” OR “Geographic Visualization” OR “Geovisualization” OR “Geographical Information System*” OR “Geographic Information System*” OR “Geofencing” OR “Geographical Distribution*” OR “Geographic Distribution*” OR “Spatial Statistic*” OR “Spatial Bayesian” OR “Spatial Hotspot*” OR “Spatial Cluster*” OR “Geographic Cluster*” OR “Geographic Hotspot*” OR “Remote Sensing” OR “Global Positioning System” OR “Spatial Pattern*” OR “Spatial Data Mining” OR “Spatial Variabilit*” OR “Spatial Heterogeneit*” OR “Geostatistic*” OR “Spatial Covariance” OR “Spatial Regression” OR “Spatial Uncertaint*” OR “Spatial Point Pattern*” OR “Kriging” OR “Cartography” OR “Spatial Decision Support System*” OR “OpenStreetMap” OR “Location-Based Services” OR “Spatial Quer*” OR “GIS” OR “Web GIS” OR “Satellite Imager*” OR “ArcGIS” OR “QGIS” OR “Risk Mapping”) AND
EHR^b^	(“EHR” OR “EMR” OR “EPR” OR “Electronic Health Record*” OR “Electronic Medical Record*” OR “Electronic Patient Record*” OR “EDW” OR “Enterprise Data Warehouse” OR “RDW” OR “Research Data Warehouse”)

^a^The selected studies that used spatial analysis of EHR data were manually excluded if they lacked patient phenotype characteristics or were not conducted based on the US data.

^b^EHR: electronic health record.

### Search Strategy

The initial search comprised 2 main categories. The first category included a broad set of key terms related to spatial analysis. The second category used the key terms associated with EHR. Henceforth, our reference to EHRs will also encompass electronic medical records (EMRs), electronic patient records (EPRs), enterprise data warehouses (EDWs), and research data warehouses (RDWs). The Boolean operator AND was applied to synthesize the 2 categories.

For PubMed/MEDLINE, Scopus, and Web of Science, we used a consistent search strategy tailored to the specific features and functionalities of each platform. We used the advanced search options available on these databases to input the key terms from Table 1. The search was conducted across titles and abstracts. For Google Scholar, due to its distinct search engine and more limited filtering options compared to the other databases, we conducted broad search queries with the same key terms. We then manually reviewed the results to identify and include relevant studies that met our criteria.

### Study Selection

The retrieved abstracts and titles were imported into Covidence systematic review software (Veritas Health Innovation), where duplicate records between original databases are automatically eliminated. Two reviewers (AM and BH) independently assessed the eligibility of the studies based on the following inclusion and exclusion criteria.

The studies were eligible for primary inclusion if they (1) were composed in English; (2) were original peer-reviewed studies; (3) used individual-level patient data derived from EHRs, EMRs, EPRs, EDWs, or RDW; and (4) incorporated at least 1 form of spatial methods. Conversely, the studies were excluded if they (1) were not peer-reviewed (eg, letters, editorials, reviews, case reports, abstracts, and grey literature), (2) solely geocoded addresses or generated basic visualizations (eg, dot map and choropleth map) without any spatial analysis, and (3) not based on the US data.

The reviewers (AM and BH) independently reviewed the full texts of all remaining studies. The studies also were excluded if they lacked phenotype characteristics. Further, we manually checked the references for all the selected studies for possible inclusion. A third reviewer (AVA) was consulted to break ties.

### Data Extraction

Upon identifying studies that satisfied all inclusion criteria, two reviewers (AM and BH) extracted the following items for each study: title, publication year, country and region, sample size, study period, spatial methodologies, and key findings from the spatial methods. Moreover, studies were assessed to identify clinical domains (including primary and secondary when applicable), health conditions or problems, and themes (including social determinants of health [SDOH], environmental factors, ecological aspects, climate, microbiome, genomics, and clinical phenotypic characteristics). Previous publications have emphasized the importance of data domain sources in phenotyping, underscoring the need for validating the created phenotype [13] and using multiple data sources. Thus, in cases where the included publications did not provide details of data sources but instead referenced previously published works, referenced publications were reviewed. Additionally, we cataloged the types of EHRs that served as the sources.

### Narrative Synthesis

There is no universally accepted classification for spatial analysis methods. In this review, we have adopted and refined a classification framework based on the study of Nazia et al [14], which initially categorized methods into frequentist and Bayesian approaches and spatial and spatiotemporal methods. This classification was further broken down into descriptive, clustering, and modeling techniques [15]. Therefore, following data extraction, the studies were categorized into the following spatial methodology classifications: descriptive, clustering, modeling (frequentist), spatiotemporal (frequentist), and Bayesian. The phenotype characteristics were extracted and recorded as free text. It should be noted that the categories were not mutually exclusive.

The quality appraisal of the studies was not feasible due to the substantial heterogeneity in spatial methodologies and health domains. The geospatial distribution of the included studies was visualized using ArcGIS Pro software (version 3.0; ESRI).

## Results

### Study Selection

**Figure 1:**
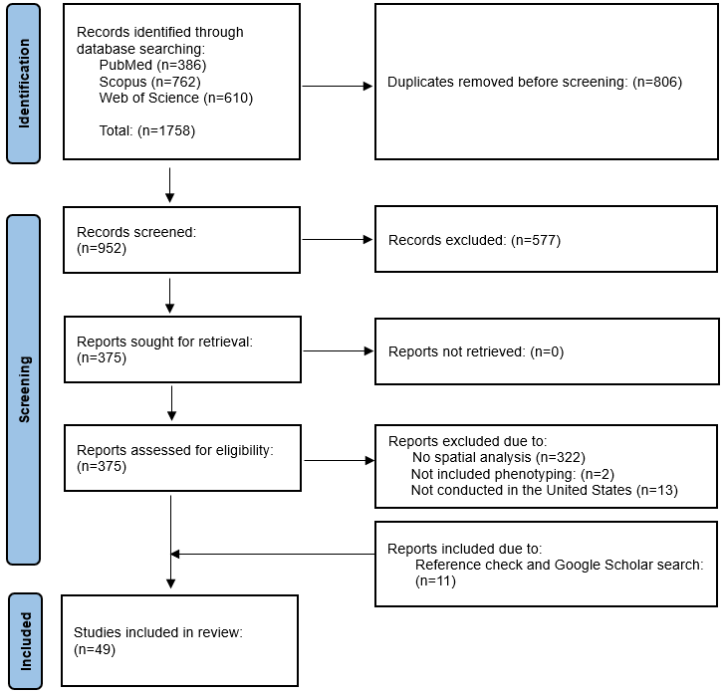
PRISMA (Preferred Reporting Items for Systematic Reviews and Meta-Analyses) study selection flowchart.

The initial search yielded 1758 references. After removing duplicate records, we identified 952 studies for abstract and title screening, from which 375 were selected for full-text review. Of these, 322 studies were excluded as they only contained geocoding or basic mapping without any spatial analysis. Additionally, 15 studies were omitted due to the absence of patient phenotype characteristics (n=2) or were not based on US data (n=13). We further manually searched references and Google Scholar and found 11 new studies that met the eligibility criteria. Therefore, 49 studies that fulfilled the inclusion criteria were retained for data extraction and synthesis. Figure 1 depicts the PRISMA flowchart for the study selection process.

### Temporal and Geographic Distribution of Studies

**Figure 2:**
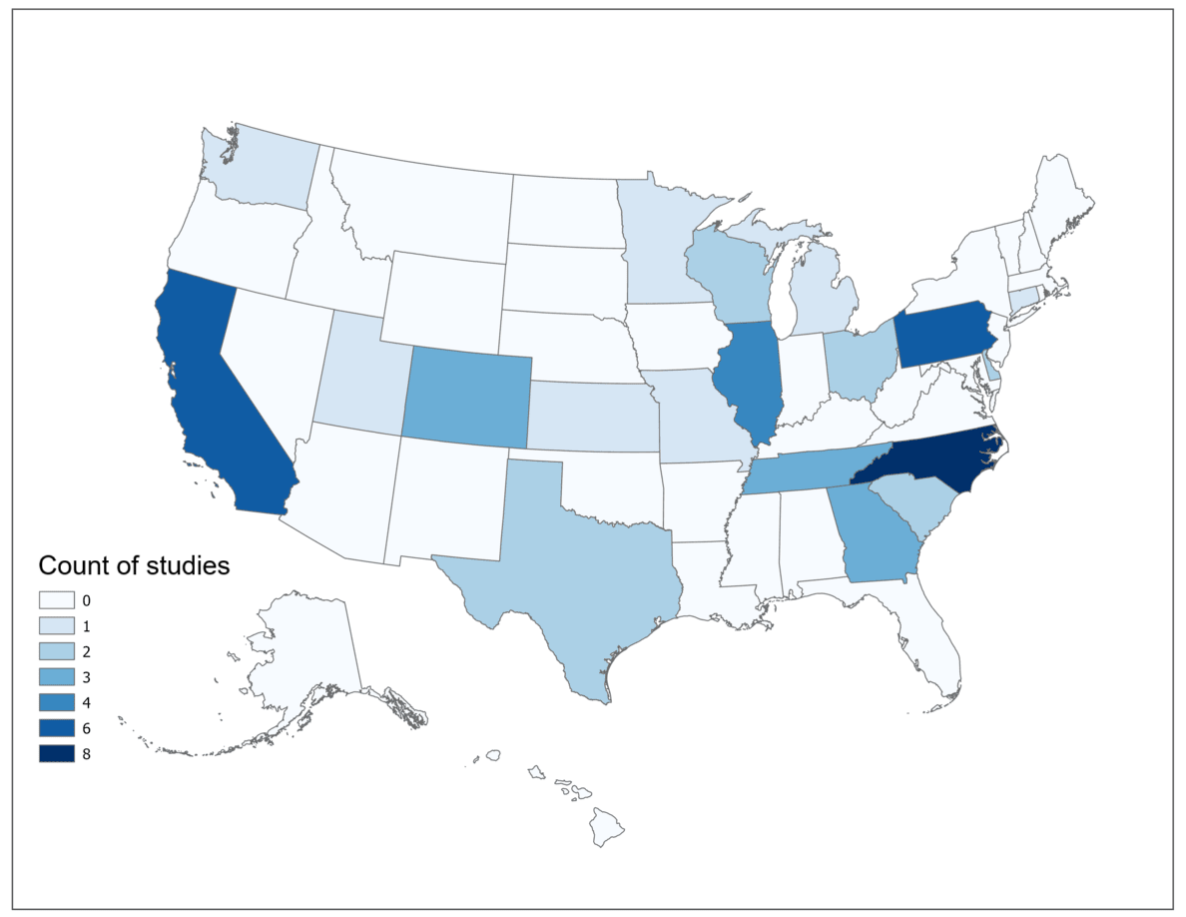
Geospatial distribution of the included studies at the state level in the United States.

Of the 49 included studies, a limited number (n=7, 14%) were published prior to 2017. The earliest study included in this study was published in 2011, and the publication frequency has experienced a significant upsurge since 2017 (n=42, 86%), likely due to increased adoption of EHR systems and growing familiarity with spatial analysis techniques among researchers. There was only one study [16] at the national level. General characteristics of the included studies are presented in Table 2. Most studies were concentrated in North Carolina (n=8, 16%), Pennsylvania (n=6, 12%), California (n=6, 12%), and Illinois (n=4, 8%). Figure 2 illustrates the geospatial distribution of studies at the state level in the United States.

**Table 2:** General characteristics of the included studies.

No.	Author	Year	Region	Sample size, n	Study period
1	Ali et al [7]	2019	Atlanta	4613	2002-2010
2	Beck et al [17]	2018	Cincinnati	24,428	2011-2016
3	Bravo et al [18]	2018	Durham	147,000	2007-2011
4	Bravo et al [19]	2019	Durham	147,351	2007-2011
5	Bravo et al [20]	2019	Durham	41,203	2007-2011
6	Brooks et al [21]	2020	Delaware	5421	2020
7	Carey et al [22]	2021	Utah	366	2006-2015
8	Casey et al [23]	2016	Pennsylvania	20,569	2006-2013
9	Chang et al [8]	2015	Wisconsin	103,690	2007-2009
10	Cobert et al [24]	2020	Durham	10,352	2013-2018
11	Davidson et al [25]	2018	Denver	21,578	2011-2012
12	DeMass et al [26]	2023	South Carolina	2195	2019-2020
13	Epstein et al [27]	2014	Los Angeles	5390	2007-2011
14	Gaudio et al [28]	2023	Tennessee	2240	2015-2021
15	Georgantopoulos et al [29]	2020	South Carolina	3736	1999-2015
16	Ghazi et al [30]	2022	Twin Cities, Minnesota	20,289	2012-2019
17	Grag et al [31]	2023	Chicago	777,994	2007-2012
18	Grunwell et al [32]	2022	Georgia	1403	2015-2020
19	Hanna-Attisha et al [33]	2016	Flint, Michigan	1473	2013-2015
20	Immergluck et al [34]	2019	Atlanta	13,938	2002-2010
21	Jilcott et al [35]	2011	Eastern North Carolina	744	2007-2008
22	Kane et al [36]	2023	Kansas and Missouri	2427	2011-2020
23	Kersten et al [37]	2018	San Francisco	47,175	2007-2011
24	Lantos et al [38]	2018	North Carolina	3527	N/A^a^
25	Lantos et al [39]	2017	Durham	3527	≤2015
26	Lê-Scherban et al [40]	2019	Philadelphia	3778	2016
27	Lieu et al [41]	2015	Northern California	154,424	2000-2011
28	Lipner et al [42]	2017	Colorado	479	2008-2015
29	Liu et al [43]	2021	Cincinnati and Houston	88,013	2011-2016
30	Mayne et al [44]	2019	Chicago	14,309	2015-2017
31	Mayne et al [45]	2018	Chicago	4748	2009-2013
32	Oyana et al [46]	2017	Memphis	28,793	2005-2015
33	Patterson and Grossman [16]	2017	Nationwide	~100 million	2003-2010
34	Pearson and Werth [47]	2019	Philadelphia	642	2000-2017
35	Samuels et al [48]	2022	New Haven	6366	2013-2017
36	Schwartz et al [49]	2011	Pennsylvania	47,769	2009-2010
37	Sharif-Askary et al [50]	2018	North Carolina	558	1998-2013
38	Sidell et al [51]	2022	Southern California	446,440	2020-2021
39	Siegel et al [52]	2022	Delaware	3449	2012-2020
40	Soares et al [6]	2017	Pennsylvania	2049	2011-2012
41	Sun et al [53]	2022	Southern California	395,927	2008-2018
42	Tabano et al [54]	2017	Denver	31,275	2009-2011
43	Wakefield et al [55]	2020	Memphis	3754	2015-2017
44	Wilson et al [56]	2022	Chicago	39,211	2014-2016
45	Winckler et al [57]	2023	Southern California	7896	2017-2019
46	Xie et al [3]	2017	Philadelphia	27,604	2011-2014
47	Xie et al [58]	2023	Washington	242,637	2015-2019
48	Zhan et al [59]	2021	Central Texas	21,923	2019
49	Zhao et al [60]	2021	Wisconsin	43,752	2007-2012

^a^Not applicable.

### Spatial Methodologies

#### Overview

Most studies focused on frequentist methods compared to the Bayesian methods. Among frequentist methods, the most prevalent category was clustering (n=29), followed by descriptive (n=12), modeling (n=6), and spatiotemporal analyses (n=2). More detailed explanations of the spatial methods used in this study are provided in Multimedia Appendix 2.

#### Descriptive Analyses

Descriptive analyses were categorized into four groups: spatial sampling (n=2), spatial overlay (n=2), proximity analysis (n=4), and spatial interpolation (n=4).

##### Spatial Sampling

A 2 SD ellipse method is used to optimize spatial sampling density. This ellipse contains almost 95% of the locations of patients and is used to ensure that the collected samples reflect the underlying spatial pattern in data, particularly when resources are limited [61]. Lantos et al [38] and Lantos et al [39] adopted this approach when sampling women who underwent cytomegalovirus antibody testing during pregnancy, especially in peripheral areas with limited subject representation.

##### Spatial Overlay

Spatial overlay integrates various spatial data sources, often maps, to represent their shared features. Wakefield et al [55] overlaid the map of major radiation treatment interruptions based on race onto the map of median household income. Their analysis implied that regions with higher income levels experienced lower rates of radiation treatment interruption. Samuels et al [48] spatially joined patient addresses to the nearest city parcels and computed an estimate of the incidence of emergency department visits for asthma for each parcel [48].

##### Proximity Analysis

Proximity analysis includes measuring distances between geographic features to identify nearby features within a defined distance or buffer zone to uncover proximity patterns [62]. Wilson et al [56] created temporal and spatial buffers to assess the correlation between individual exposure to violent crime and blood pressure. Schwartz et al [49] evaluated the associations between environmental factors and BMI within a 0.5-mile network buffer from the place of residence. Casey et al [23] investigated the associations between prenatal residential greenness and birth outcomes within 250-m and 1250-m buffers. Using a geographic information system service area network analysis, Jilcott et al [35] examined BMI percentile and proximity to fast-food and pizza establishments among adolescents within 0.25-mile Euclidean and network buffer zones.

##### Spatial Interpolation

Ordinary Kriging is one of the most widely used spatial interpolation techniques that leverages the spatial autocorrelation structure of observed locations to estimate values at unmeasured locations [63]. Hanna-Attisha et al [33] applied ordinary Kriging with a spherical semivariogram model based on observations of the children’s elevated blood lead level geocoded to the home address to visualize blood lead level variations before and after water source changes. Mayne et al [44] interpolated the levels of neighborhood physical disorder based on an exponential variogram. Patterson and Grossman [16] demonstrated spatial variations for the incidence rates of each *International Classification of Diseases, Ninth Revision* diagnostic code based on an exponential variogram. Sun et al [53] estimated monthly average concentrations of fine particulate matter to investigate the associations between air pollution exposure during pregnancy and gestational diabetes mellitus.

#### Spatial Clustering

##### Overview

Spatial clustering techniques assess whether health outcomes are random, uniform, or clustered and pinpoint the locations of clusters [64]. Spatial clustering was the most widely used category (n=29) among all studied categories. Moran *I* clustering and cluster detection were the most frequent techniques (n=10), followed by kernel/point density estimation (n=5), spatial scan statistics (n=4), and Getis-Ord Gi* statistics (n=4).

##### Kernel/Point Density Estimation

Kernel density estimation generates a smooth surface to visualize areas of the most significant spatial intensity by calculating a distance-weighted count of events within a specified radius per unit area [65]. Several studies adopted kernel density estimation to analyze patterns, including cholera hospitalization [58], comparison of the spatial intensity of chronic kidney disease with nonchronic kidney disease patients [30], and comparison of the spatial intensity of breast cancer and nonbreast cancer [52]. Using the point density function, Beck et al [17] pinpointed hotspots of inpatient bed-day rates within a 2-mile radius of a medical center, and Kane et al [36] estimated the number of participants per square mile.

##### Global and Local Moran I

Global Moran *I* (GMI) evaluates the overall pattern for spatial autocorrelation [66] by inferring if a variable is spatially clustered or overdispersed versus being randomly distributed under the null hypothesis [66]. Local Moran *I* (LISA) is used to locate statistically significant clusters including hotspots, cold spots, and outliers [67]. GMI has been adopted to analyze spatial clustering of health outcomes including gestational diabetes mellitus [53], day-of-surgery cancellation [43], obesity [54], and COVID-19 [51]. All exhibited clustered patterns. Xie et al [58] analyzed 3 groups: depression, obesity, and comorbid cases, confirmed clustering for all outcomes, and identified spatial clusters and outliers. Pearson and Werth [47] found random distributions for dermatomyositis (DM) and subtypes, classic DM, and clinically amyopathic DM. Meanwhile, Davidson et al [25] pinpointed clusters with higher or lower depression prevalence, and Winckler et al [57] identified a cluster of low use of acute pediatric mental health interventions in less-densely populated rural border areas.

GMI and semivariograms or variograms can also identify spatial autocorrelation in model residuals. If detected, the models are adjusted accordingly to avoid biased estimates. For example, Lipner et al [42] modeled nontuberculous mycobacteria disease, shifting the use from a nonspatial Bayesian model to a spatial model when spatial autocorrelation was found in residuals. Similarly, Georgantopoulos et al [29] incorporated spatial random effects into a prostate cancer model due to significant autocorrelation in the residuals. Sharif-Askary et al [50] used variograms to assess spatial dependency in cleft lip or palate, leading to a geostatistical model over standard logistic regression. Conversely, Casey et al [23] found no spatial autocorrelation in nonspatial model residuals.

The bivariate GMI quantifies the overall spatial dependence between two distinct variables (positive value indicates high values of one variable are surrounded by high values of the other or low values are surrounded by low values, while negative value implies high values of one variable are surrounded by low values of the other) [68]. Bivariate LISA assesses the relationship between the two variables at the local level. Pearson and Werth [47] used bivariate GMI for the prevalence of DM, classic DM, and clinically amyopathic DM with airborne toxics but found no overall spatial dependencies. However, bivariate LISA identified local dependencies at the zip code level. Garg et al [31] applied bivariate GMI and found significant overall associations between longer (average) distances to the nearest supermarket and higher incidence of diabetes, and bivariate LISA identified significant “high-high” relationships at the zip code level. Gaudio et al [28] used bivariate LISA and found no local association between radiation therapy interruption and social vulnerability index at the zip code level.

##### Getis-Ord Gi*

The Getis-Ord Gi* statistic identifies high- or low-value clusters (hotspots and cold spots) by assessing deviations of health outcomes at locations from the average within a defined neighborhood [69]. Lê-Scherban et al [40] measured racial residential segregation by examining the deviations in the African American residents in each census tract from the mean of neighboring tracts. Similarly, Mayne et al [45] measured racial residential segregation for the percentage of non-Hispanic Black residents. Ali et al [7] identified significant community-onset methicillin-resistant Staphylococcus aureus (CO-MRSA) hotspots with distinct patterns between cases and controls. Kersten et al [37] detected the high- and low-value clusters for the child opportunity index and median household income.

##### Spatial Scan Statistics

The spatial scan statistics technique identifies high- and low-risk clusters and estimates their relative risks [70]. It also can incorporate covariates to characterize underlying patterns [71]. Lipner et al [42] found that people living in zip codes within the primary cluster had an almost 2.5 times greater risk of nontuberculous mycobacteria disease. Lieu et al [41] identified clusters of underimmunization and vaccine refusal among children, with rates ranging from 18% to 23% inside the clusters compared to 11% outside.

The technique can also pinpoint cold spots. Brooks et al [21] identified areas with significantly lower COVID-19 testing than expected, indicating a need for interventions. Zhan et al [59] observed significantly low rates of up-to-date colorectal cancer screening.

#### Spatial Modeling (Frequentist)

Among the included studies, the generalized additive models (GAMs) emerged as the most frequently used spatial models. GAMs can account for spatial autocorrelation by incorporating smooth functions (such as thin-plate regression) of spatial coordinates [72], allowing the estimate of geographic variation with or without covariate adjustments. GAMs were used to identify the spatial variabilities in asthma prevalence [3,8] and cytomegalovirus [38,39], although such variations often diminished when adjusted for demographic factors such as race and age. Less commonly used geospatial models were generalized linear mixed effects [51] and spatial error [43] models.

#### Spatiotemporal Analysis

Only 2 studies explored spatiotemporal patterns, and no spatiotemporal modeling was conducted. Oyana et al [46] used space-time scan statistics to study the spatiotemporal patterns of childhood asthma and found a significant frequency increase (2009-2013) and a rising trend from 4 to 16 per 1000 children (2005-2015). Ali et al [7] used the space-time cube tool and emerging hotspot analysis to analyze the spatial-temporal trends and evolving patterns of CO-MRSA from 2002 to 2010. They identified several types of space-time hotspots of CO-MRSA including new, consecutive, intensifying, sporadic, and oscillating hotspots.

#### Bayesian Analysis

The studies using Bayesian methods were categorized into empirical Bayes smoothing (n=5) and Bayesian modeling (n=6).]

##### Empirical Bayes Smoothing

The empirical Bayes smoothing was used by Lê-Scherban et al [40], Liu et al [43], Tabano et al [54], and Xie et al [58] to stabilize estimated rates in areas with limited data points by borrowing information from the overall population [73]. Zhao et al [60] used nonparametric kernel smoothing to estimate the prevalence of childhood obesity in areas with sparse observations (n<20 individuals) [60].

##### Bayesian Modeling

Bayesian modeling can account for spatial and temporal dependencies and quantify uncertainty by specifying prior distributions [74]. Among the studies, the conditional autoregressive (CAR) prior emerged as the most used, with 2 variants: intrinsic and multivariate CAR. Intrinsic CAR was used to assess the spatial variations in diabetes in relationship with racial isolation [18], hypertension related to racial isolation [19], and type 2 diabetes mellitus with the built environment [20]. Multivariate CAR was used to identify areas with higher or lower-than-expected prostate cancer while controlling for risk factors [29]. Moreover, hierarchical Bayesian that can incorporate hierarchical structures for modeling [75] was used to investigate spatial distributions of patients admitted for drug-related reasons concerning the area deprivation index [24]. Bayesian negative binomial hurdle models that can account for excessive zeros and overdispersion were used to examine spatial variation between patient responses to the questions concerning unhealthy home environments and the mean number of emergency department visits after screening [26].

### Phenotyping

#### Clinical Domain Characteristics and Themes

The largest category of studies was classified under the infectious disease (n=7), endocrinology (n=7), and oncology (n=6) domains. Additionally, 19 studies had a pediatric domain or focus, as noted with an additional column in Table 3. Maternal and newborn care was classified as its own domain (n=8), but it overlapped with other domains such as nephrology, endocrinology, and infectious disease.

**Table 3:** Clinical domains and condition or problem of focus for each publication.

Condition by clinical domain^a^		Secondary clinical domain^b^	Pediatric population involved
**Pediatric**			
	DoSC^c^ [43]	—^d^	✓
	EBLL^e^ [33]	—	✓
	Disparities in inpatient bed-day rates [17]	—	✓
**Maternal and newborn care**			
	Under immunization; vaccine refusal [41]	—	✓
	Preterm birth; small for gestational age; hypertensive disorder of pregnancy [44]	—	
	Preterm birth; small for gestational age; low birth weight; low Apgar score [23]	—	
	Hypertension [56]	—	
	Hypertension [19]	—	
	Hypertension; diabetes [40]	Endocrinology	
	Hypertension; diabetes; CKD^f^ [31]	Endocrine; nephrology	
	Hypertension, disorder of pregnancy [45]	Maternal and newborn care	
**Endocrinology**			
	GDM^g^ [53]	Maternal and newborn care	
	T2DM^h^ [18]	—	
	T2DM [20]	—	
	Obesity [54]	—	
	Obesity [49]	—	✓
	Obesity [35]	—	✓
	Obesity [60]	—	✓
	Obesity; depression [58]	Psychiatry	
**Psychiatry**			
	Acute pediatric mental health interventions or services [57]	—	✓
	Depression [25]	—	
	Telemedicine use in developmental-behavioral pediatrics [6]	—	✓
	Drug overdoses [24]	Emergency medicine	
**Emergency medicine**			
	Disparities in pediatric acute care visit frequency and diagnoses [37]	—	✓
	Disparities in use of PICU^i^ [27]	—	✓
	Emergency department use [26]	—	
**Pulmonary**			
	Asthma, emergency department asthma visits [48]	Emergency medicine	
	Asthma [32]	—	✓
	Asthma [46]	—	✓
	Asthma [3]	—	
	Asthma [8]	—	
**Infectious disease**			
	Coccidioidomycosis [22]	Pulmonary	
	Community-associated MRSA^j^ [34]	—	✓
	Community-onset-MRSA [7]	—	✓
	COVID-19 [21]	—	
	COVID-19 [51]	—	
	CMV^k^ [39]	Maternal and newborn care	✓
	CMV [38]	—	✓
	Nontuberculous mycobacterial infection [42]	—	
**Oncology**			
	RTI^l^ [55]	—	
	RTI [28]	—	
	Colorectal cancer screening [59]	—	
	Prostate cancer [29]	—	
	TNBC^m^ [52]	—	
	Disparities in genomic answers for kids (GA4K) [36]	—	✓
**Maxillofacial**			
	Cleft lip or palate [50]	—	✓
**Nephrology**			
	CKD [30]	—	
**Rheumatology**			
	Dermatomyositis [47]	Neurology; dermatology	
**All domains**			
	Geospatial variation of disease incidence [16]	—	

^a^Condition or problem of focus column displays the general condition of the study and may not directly correspond to the phenotype.

^b^Publications with more than 1 clinical domain and those with a pediatric component are noted as such.

^c^DoSC: day-of-surgery cancellation.

^d^Not applicable.

^e^EBLL: elevated blood lead levels.

^f^CKD: chronic kidney disease.

^g^GDM: gestational diabetes mellitus.

^h^T2DM: diabetes mellitus, type 2.

^i^PICU: pediatric intensive care unit.

^j^MRSA: methicillin-resistant Staphylococcus aureus.

^k^CMV: cytomegalovirus.

^l^RTI: radiation treatment interruption.

^m^TNBC: triple-negative breast cancer.

The relationship between the clinical domains and the “conditions or problems of focus” in each study was examined (Table 3). In some cases, direct correspondence was observed, while in other instances, the “condition or problems of focus” differed from the phenotype of the patient cohort. In many studies, one or more overlapping domains were observed (eg, rheumatology, neurology, and dermatology for the study of DM). Asthma (n=5), hypertension (n=5), and diabetes (n=4) were studied most frequently. Three studies did not focus on any health condition but rather on examining disparities in either a data source or a specific domain or cohort (eg, disparities in the use of pediatric intensive care units).

Every study was attributed to at least one prominent theme, with the possibility of multiple themes. SDOH themes were prevalent in many studies. To organize and present this information, we used the domains defined by the Healthy People 2030 framework [76]. There are 5 domains in the SDOH framework (Table 4), with the corresponding counts of these domains being seen as themes of the studies. Most studies had 1 or more SDOH themes (n=42). Many studies focused either on all the domains or SDOH holistically without particular focus on any specific domain (n=32). However, some studies contained prominent themes that were not directly related to SDOH, which were phenotypic features (n=4), followed by environmental (n=3), and ecological (n=2), with climate, genomics, and microbiome, each contributing one study.

**Table 4:** SDOH^a^ themes examined within the framework of Healthy People 2030 SDOH domains [76].

Labels and SDOH domains		Counts, n
**SDOH 1**		
	Economic stability (employment, food insecurity, housing instability, poverty)	2
**SDOH 2**		
	Education access and quality (early childhood development and education, enrollment in higher education, high school graduation, language, and literacy)	N/A^b^
**SDOH 3**		
	Health access and quality (access to health services, access to primary care, health literacy)	5
**SDOH 4**		
	Neighborhood and built environment (access to foods that support healthy dietary patterns, crime and violence, environmental conditions, quality of housing)	14
**SDOH 5**		
	Social and community context (civic participation, discrimination, incarceration, social cohesion)	5
**All 5 SDOH domains or SDOH as a whole**		36
**Non-SDOH focus**		8

^a^SDOH: social determinants of health.

^b^Not applicable.

#### Clinical Phenotype Features

For each publication, clinical phenotype definitions were extracted (Multimedia Appendix 3). In almost all studies, phenotype definitions included demographic details such as patient age, race, and gender, along with some diagnostic characteristics (eg, asthma diagnosis). Only a limited number of phenotypes were observed to be validated (n=8). The most frequently observed method for phenotype validation was a manual chart review of all matches or a sample of matched charts. None of the studies with chart review as a validation method shared information on the match rate. Additionally, only two studies [20,58] were observed to use validated eMERGE Network computable phenotypes from the Phenotype Knowledgebase [77-79].

## Discussion

### Principal Findings

This systematic review is the first comprehensive investigation of spatial methodologies within EHR-derived data in the United States. The findings reveal that a considerable portion of studies predominantly focus on basic mapping or geocoding, with a limited use of advanced spatial analysis methods. Spatial clustering and descriptive analysis were the most used methods, while space-time modeling, either frequentist or Bayesian, was not widely applied. The diverse use of spatial analysis for EHR-derived data in different health domains highlights the potential to incorporate spatial methods to enhance the context of individual patients for future biomedical research. We found limited use of EHR-derived data for spatial analysis, probably due to the challenge of safeguarding patient privacy. Address data, crucial for spatial analysis, is highly confidential and often restricted from sharing. Researchers and institutions often use geographic masking techniques [6,80] to balance data use and privacy protection by altering the precise geographic coordinates while preserving the overall spatial characteristics of data. Encouraging the adoption of spatial analysis could promote biomedical knowledge sharing and collaboration.

The use of EHRs data for spatial analysis can present several challenges, particularly in accurately geocoding patient addresses. Issues, such as address formatting errors, incomplete or outdated addresses, and potential inaccuracies in geocoding services, can influence the outcome of spatial analysis [81]. Advanced geocoding algorithms and manual verification processes can mitigate these issues. For instance, Goldberg et al [82] developed a web-based system for rapid manual intervention of previously geocoded data, significantly improving the match rate and quality of individual geocodes with minimal time and effort. Additionally, when addresses are only available at the zip code level, additional nuances arise as zip code boundaries are often not well-defined and can change over time [83]. Spatial smoothing techniques and zip code centroids can mitigate some of these challenges. We recommend standardizing address formats before geocoding (using tools like the US Postal Service address verification), using advanced geocoding services, leveraging higher-resolution geographical data when possible, and integrating multiple spatial scales to enhance the accuracy and reliability of spatial analysis using EHRs data.

We acknowledge that not all patient phenotypes are inherently suited for spatial analysis, and integrating genomics, imaging, and clinical notes phenotypes can be particularly challenging. However, evidence suggests that spatial techniques can provide valuable insights even in these areas where their application may initially appear challenging. For instance, Baker et al [84] demonstrated the effectiveness of spatial analysis in genomics by combining single-nucleotide polymorphism genotyping with geospatial K-function analysis. Their study of typhoid in Nepal found significant geographic clustering of cases. Canino [85] developed a robust framework that integrated biological data with geographic information from EMRs. Their system identified correlations between patient profiles and geographic factors such as environmental exposures related to pollution. Future interdisciplinary studies can explore developing frameworks that integrate genomics or notes with geospatial datasets to reveal complex relationships and patterns.

The application of spatiotemporal analysis of EHR-derived data was mainly limited to exploring spatiotemporal clusters with no spatiotemporal modeling. This might be due to the technical expertise required for analysis, data complexity, availability of longitudinal data, and computational challenges. The Bayesian framework offers a more adaptable framework to handle complex spatial and temporal dependencies, control confounding variables [86], and incorporate prior information, such as existing medical literature and expert opinions, resulting in more interpretable results [87,88]. Moreover, spatiotemporal Bayesian modeling can aid in understanding disease trends and progressions, seasonality, and long-term shifts at the local levels [89]. Bayesian modeling can also account for uncertainty in parameter estimates and predictions to assess the reliability of findings before implementing interventions [90]. Thus, future research should delve into spatial and spatiotemporal modeling, focusing on Bayesian approaches. Moreover, ignoring spatial dependence in modeling can bias parameter estimates [9,91,92]. Additional state-of-the-art methods, such as space-time autoregressive models and generalized additive models for location scale and shape, also provide flexibility in modeling complex relationships. Spatiotemporal point process models also contribute by analyzing the distribution of health events and underlying states over space and time.

Among the health conditions studied, chronic and infectious diseases emerged as the most frequently investigated domains compared to others. This disparity may be attributed to the pressing public health concerns posed by diseases with immediate impacts that often attract more funding and resources for research initiatives [93,94]. The historically high mortality rates of these conditions likely led to continuous research. Furthermore, the nature of spatial contamination and the spread of infectious diseases has historically driven the development of spatial analysis for clinical purposes, exemplified by John Snow’s seminal cholera investigation. Surprisingly, despite the plethora of funding in cancer research, we only found a small number of studies within the cancer domain, which may likewise be attributed to and indicative of the pressing needs of other domains such as infectious disease.

We observed recurring and prominent themes related to the SDOH. This emphasis may result from the growing maturity and increased awareness within the biomedical informatics community regarding the significant influence of social, economic, and environmental factors on health outcomes. Understanding the roles of SDOH in health disparities will likely lead to the implementation of integrative health interventions that address the needs of individuals affected by these health disparities. These interventions can likewise be enhanced by incorporating spatial perspectives.

Another missed opportunity is the limited use of computable phenotypes—automated algorithms designed for characterizing diseases and enrolling patients in studies. Most studies primarily depended on the manual application of inclusion and exclusion criteria to define phenotypes. While this method may be suitable in certain scenarios, it often necessitates greater depth and granularity to consistently and accurately capture the intended patient cohorts. The accuracy and precision of the manual approach can vary depending on the data sources and clinical domains. Notably, only 2 of the studies in this review used computable phenotypes, indicating a limited adoption of this essential and potentially transformative approach, highlighting a noteworthy area for growth. Furthermore, only 5 studies carried out any form of chart review validation. Validation methods, including chart reviews, genetic markers, and clinical variables, are indispensable in phenotyping to guarantee the accurate characterization of the desired cohorts. This applies even to computable phenotypes within specific medical domains [95].

### Limitations

This study has several main limitations. First, we only considered English-language studies, possibly introducing language bias. Additionally, selection bias is possible due to database availability. However, we mitigated these limitations by searching Google Scholar and conducting backward reference checking to identify relevant studies that might yet be identified through our initial search strategy. Finally, we used a query search strategy with limited keywords, which inherently restricted the scope of studies we could retrieve, potentially omitting studies that did not use these specific terms in their abstract or title.

Our rationale to focus exclusively on US data was driven by our familiarity with the reliability and availability of EHR-based systems within the country. Moreover, we recognize that spatial analyses of health data in regions, such as Europe, Asia, Australia, and Canada, use different terminologies and labels for their systems, which might not align with our search terms for EHRs or EMRs. For instance, Canada’s national administrative databases and electronic discharge records could encompass significant work not captured by our key terms, a situation that can be generalized to other countries. To avoid inconsistencies arising from varying data labeling and storage systems across different regions, we opted to concentrate on the United States. Nevertheless, future research should endeavor to include and explore contributions from these regions to provide a more comprehensive understanding of emerging trends in spatial analysis in characterizing patient phenotypes.

### Conclusions

This systematic review provided a comprehensive overview of the current use of spatial analysis in EHR-based research in the United States and underscored the pivotal role that spatial analysis can play in clinical decision support and interventions. The use of EHR-derived spatial analysis is on an upward trajectory, parallel with the widespread adoption of EHR systems. The volume of studies on this topic is anticipated to continue to grow. The primary health outcomes investigated were asthma, hypertension, and diabetes. Notably, patient phenotypes involving genomics, imaging, and notes that are notoriously high-dimensional and add to the computational intensity of spatial methods were limited. This review also highlighted the need for additional exploration of spatial analysis techniques, including but not limited to spatiotemporal Bayesian analysis and modeling, particularly in computable phenotypes or patient phenotypes involving genomics, imaging, and notes.

## Appendix

Multimedia Appendix 1 PRISMA (Preferred Reporting Items for Systematic Reviews and Meta-Analyses) checklist.

Multimedia Appendix 2 Detailed descriptions of the spatial methods used.

Multimedia Appendix 3 Clinical phenotype definitions and spatial method used for each publication.

## Abbreviations

CAR: conditional autoregressive
CO-MRSA: community-onset methicillin-resistant Staphylococcus aureus
DM: dermatomyositis
EDW: enterprise data warehouse
EHR: electronic health record
EMR: electronic medical record
EPR: electronic patient record
GAM: generalized additive model
GMI: global Moran I
LISA: local Moran I
PRISMA: Preferred Reporting Items for Systematic Reviews and Meta-Analyses
SDOH: social determinants of health
