# Supplementary material for: Application of Spatial Analysis on Electronic Health Records to Characterize Patient Phenotypes: Systematic Review
Source: JMIR Med Inform. 2024 Oct 15;12:e56343. doi: 10.2196/56343 (PMC11522649; doi:10.2196/56343)
Supplement: Multimedia Appendix 2 [file medinform_v12i1e56343_app2.docx]

**Two standard deviation ellipses**

The two standard deviation ellipse can measure the center, dispersion, and direction trend of the spatial distribution of geographical features typically covering about 95% of the features under a normal distribution (Liu et al., 2020):

Weighted average center:

$\bar{X}_{w}=\frac{\sum_{i=1}^{n} w_{i}x_{i}}{\sum_{i=}^{n} w_{i}}$ ; $\bar{Y}_{w}=\frac{\sum_{i=1}^{n} w_{i}y_{i}}{\sum_{i=}^{n} w_{i}}$

Elliptic direction:

$$tan\theta=\frac{A+B}{C}$$

$$A=\left( \sum_{i=1}^{n} w_{i}^{2}\tilde{x}_{i}^{2}-\sum_{i=1}^{n} w_{i}^{2}\tilde{y}_{i}^{2} \right)$$

$$B=\sqrt{\left( \sum_{i=1}^{n} w_{i}^{2}\tilde{x}_{i}^{2}-\sum_{i=1}^{n} w_{i}^{2}\tilde{y}_{i}^{2} \right)+4\sum_{i=1}^{n} w_{i}^{2}\tilde{x}_{i}^{2}\tilde{y}_{i}^{2}}$$

$$C=2\sum_{i=1}^{n} w_{i}^{2}\tilde{x}_{i}\tilde{y}_{i}$$

Standard deviation of the X-axis:

$$\sigma_{x}=\sqrt{\frac{\sum_{i=1}^{n} \left( w_{i}\tilde{x}_{i}\cos\theta-w_{i}\tilde{y}_{i}\sin\theta\right)^{2}}{\sum_{i=1}^{n} w_{i}^{2}}}$$

Standard deviation of the Y-axis:

$$\sigma_{y}=\sqrt{\frac{\sum_{i=1}^{n} \left( w_{i}\tilde{x}_{i}\sin\theta-w_{i}\tilde{y}_{i}\cos\theta\right)^{2}}{\sum_{i=1}^{n} w_{i}^{2}}}$$

Here, $\left( x_{i},y_{i} \right)$ denotes the spatial coordinate of the feature $i$; $w_{i}$represents the weight at the spatial element $i$. ($\bar{X}_{w},\bar{Y}_{w}$) indicates the weighted average center of the spatial data set. $\theta$ is the azimuth angle of ellipse, while $\tilde{x}_{i}$ and $\tilde{y}_{i}$ show the deviation of coordinates from the average center. $\sigma_{x}$ and $\sigma_{y}$ denote the standard deviation along the X and Y axes.

**Spatial Interpolation**

Ordinary Kriging is a geostatistical interpolation method that estimates values at unsampled locations based on known data points, assuming a constant mean across the study area. It uses the spatial autocorrelation quantified by semi variogram to calculate optimal weights for interpolation. The interpolated value at each point is calculated based on (Khan et al., 2023):

$$Z^{*}\left( x_{0} \right)=\sum_{i=1}^{n} \lambda_{i} Z\left( x_{i} \right)$$

Where $Z^{*}\left( x_{0} \right)$ represents the predicted value at ​$x_{0}$; $Z\left( x_{i} \right)$ are the values of nearby points; $\lambda_{i}$ represents the weights or how much each nearby point affects the prediction which is calculated from semi variogram as follows:

$$\gamma\left( h \right)=\frac{1}{2N\left( h \right)} \sum_{i=1}^{N\left( h \right)} \left( Z\left( x_{i} \right)-Z\left( x_{i}+h \right) \right)^{2}$$

Where $\gamma\left( h \right)$ is the semi variogram; $h$ is the distance between points; N(h) is the number of sample pairs.

**Kernel Density estimation**

The kernel density estimation (KDE) method centers on each sample point and calculates the density for each grid unit within a specified range (circle with radius h). The density increases as the distance from the sample point's center decreases (Liu et al., 2020). The KDE is calculated as follows:

$$f\left( s \right)=\sum_{i=1}^{n} \frac{1}{h^{2}}k\left( \frac{s-s_{i}}{h} \right)$$

Where $f\left( s \right)$ represents the estimated kernel density at the position $s$; $k\left( \frac{s-s_{i}}{h} \right)$ is the kernel function; ($s-s_{i}$) denotes the distance between the estimated point $s$ and the sample point $s_{i}$; $n$ is the number of points within a specified range; $h$ is the radius of circle.

**Global Moran’s *I***

Global Moran’s *I* is used to examine whether the existing pattern is random, clustered, or dispersed. The null hypothesis assumes complete spatial randomness (Mollalo et al., 2021). The statistic is defined as (Bivand & Wong, 2018):

$I=\frac{n\sum_{i=1}^{n} \sum_{j=1}^{n} w_{ij} \left( x_{i}-\bar{x} \right)\left( x_{j}-\bar{x} \right)}{\sum_{i=1}^{n} \sum_{j=1}^{n} w_{ij} \sum_{i=1}^{n} \sum_{j=1}^{n} w_{ij} \left( x_{i}-\bar{x} \right)^{2}}$

Where $x_{i}$ and $x_{j}$ are attribute values for location $i$ and location$j$; $\bar{x}$ is the mean attribute value; $w_{ij}$ is the spatial contiguity weight matrix, $w_{ij}$=1 when two locations are adjacent and $w_{ij}$=0 otherwise; and $n$ is the total number of locations. The coefficient of $I$ ranges between -1 and +1. The value approaching +1 indicates clustered pattern (positive autocorrelation), the value approaching -1 suggests dispersed distribution (negative autocorrelation), and the value close to 0 denotes the random distribution.

**Local Moran’s I**

Using the same notation as Global Moran’s *I*, Anselin Local Moran’s $I$or LISA is used to identify the concentrations of low and high attribute values for location $i$ as (Anselin, 1995):

$$I_{i}=\frac{\left( x_{i}-\bar{x} \right)}{S_{i}^{2}}\sum_{j=1}^{n} w_{ij} \left( x_{j}-\bar{x} \right)$$

$$S_{i}^{2}=\frac{\sum_{j=1, i\neq j}^{n} w_{ij}}{n-1}-\bar{x}^{2}$$

A positive value for $I_{i}$ either shows a location with high attribute values is surrounded by locations with high attribute values (High-High cluster) or a location with low attribute values is surrounded by locations with low attribute values (Low-Low cluster). A negative value for $I_{i}$ suggests that a location with high attribute values is surrounded by locations with low attribute values and vice versa (Outlier).

**Getis-Ord Gi***

Getis-Ord Gi* statistic is used to identify hot spots or cold spots values as (Getis & Ord, 1992):

$G_{i}^{*}=\frac{\sum_{j=1}^{n} w_{ij}x_{j}- \bar{x}\sum_{j=1}^{n} w_{ij}}{S \sqrt{\frac{[n\sum_{j=1}^{n} w_{ij}^{2}-\left( \sum_{j=1}^{n} w_{ij} \right)^{2}]}{n-1}}}$

Where $\bar{x}$and $S$ represent the mean and standard deviation of attribute value for location $i$, respectively. A significant positive Gi* value above the expected indicates a hot spot, while a negative significant value below the expected indicates a cold spot.

**Spatial scan statistics**

Spatial scan statistics are used to identify clusters of cases in a geographic area by employing a scanning circular window. The Log-Likelihood Ratio (LLR) is a measure used to evaluate the significance of identified clusters. It compares the observed number of cases within a circular window to the expected number of cases under the null hypothesis (random distribution). If the LLR of a cluster is among the highest values compared to those obtained in the simulation, it is considered statistically significant (Kulldorff, 1997).

$$LLR=\frac{\left( \frac{n_{Z}}{\mu_{Z}} \right)^{n_{Z}}\left( \frac{N-n_{Z}}{N-\mu_{Z}} \right)^{N-n_{Z}}}{\left( \frac{N}{\mu_{T}} \right)^{N}}$$

Where $n_{Z}$ is the number of cases within the circular window Z; $\mu_{Z}$ is the expected number of cases within the circular window Z; $N$is the total number of observed cases for the entire study area; $\mu_{T}$ is the total number of expected cases across the study area.

The relative risk is the risk of cases within the scanning window compared to those outside the window and is calculated for each cluster as:

$$RR=\frac{\frac{n_{Z}}{\mu_{Z}}}{\frac{\left( n_{G}-n_{Z} \right)}{\left( \mu_{G}-\mu_{Z} \right)}}$$

**Space-time scan statistics**

Space-time scan statistics are used to identify the spatial and temporal clusters of cases by employing a scanning cylindrical window. The base of the cylinder represents space, while the height represents time. The window with the maximum likelihood is considered the most likely cluster, and secondary clusters are also presented if they are statistically significant. Log-likelihood ratios (LLR) of different windows are calculated based on the Poisson distribution and tested using the Monte Carlo method to determine the statistical significance of space-time clusters. If the LLR of a cluster is among the highest values compared to those obtained in the simulation, it is considered statistically significant. The LLR of the scanning window is calculated as (Ma et al., 2021):

$$LLR=\frac{\left( \frac{n_{Z}}{\mu_{Z}} \right)^{n_{Z}}\left( \frac{N-n_{Z}}{N-\mu_{Z}} \right)^{N-n_{Z}}}{\left( \frac{N}{\mu_{T}} \right)^{N}}$$

Where $n_{Z}$ is the number of cases within the cylinder Z; $\mu_{Z}$ is the expected number of cases within the cylinder Z; $N$is the observed number of cases for the entire study area and time; $\mu_{T}$ is the total number of expected cases in the study area across all time periods.

The relative risk is the risk of cases within the scanning window compared to those outside the window and is calculated for each cluster as:

$$RR=\frac{\frac{n_{Z}}{\mu_{Z}}}{\frac{\left( n_{G}-n_{Z} \right)}{\left( \mu_{G}-\mu_{Z} \right)}}$$

**Bayesian Spatial Modeling**

Let the observed outcomes $Y_{i}$ in regions $i=1,2,\ldots,n$ follow a normal distribution. The BYM (Besag-York-Mollié) model is defined as follows (Moraga, 2023):

$$Y_{i}\sim Normal \left( \mu_{i},\sigma^{2} \right), i=1, 2,\ldots, n$$

Where

$$\mu_{i}=z_{i}\beta+u_{i}+v_{i}$$

In this specification, the fixed effects $z_{i}\beta$ are expressed as a linear combination of an intercept and $p$ covariates for each area $i$. Here, $zi=(1,z_{i1},z_{i2},\ldots,z_{ip})$ and $\beta=(\beta_{0},\beta_{1},..,\beta_{P})$. The model incorporates a spatial random effect $u_{i}$ to account for spatial dependence, indicating that nearby areas exhibit similar outcomes. Additionally, an unstructured random component $v_{i}$ models uncorrelated noise. The $u_{i}$ is modeled using an intrinsic conditional autoregressive (ICAR) model, which smooths the data based on a defined neighborhood structure. Specifically:

$$u_{i}|u_{-i} \sim Normal(\bar{u}_{\delta_{i}}, \sigma_{u}^{2}/n_{\delta_{i}})$$

Where$\delta_{i}$ is the set of neighbors of region $i$; $\bar{u}_{\delta_{i}}$ represents the average of the spatial random effects $u_{j}$ for the neighbours of region $i$; $n_{\delta_{i}}$ denotes the number of neighbors for region $i$.

The unstructured component $v_{i}$ is modeled as independent and identically distributed normal variables with zero mean and variance $\sigma_{v}^{2}$ as:

$$v_{i}\sim Normal(0, \sigma_{v}^{2})$$

**References**

Anselin, L. (1995). Local indicators of spatial association—LISA. *Geographical analysis*, *27*(2), 93-115.

Bivand, R. S., & Wong, D. W. (2018). Comparing implementations of global and local indicators of spatial association. *Test*, *27*(3), 716-748.

Getis, A., & Ord, J. K. (1992). The analysis of spatial association by use of distance statistics. *Geographical analysis*, *24*(3), 189-206.

Khan, M., Almazah, M. M., EIlahi, A., Niaz, R., Al-Rezami, A., & Zaman, B. (2023). Spatial interpolation of water quality index based on Ordinary kriging and Universal kriging. *Geomatics, Natural Hazards and Risk*, *14*(1), 2190853.

Kulldorff, M. (1997). A spatial scan statistic. *Communications in Statistics-Theory and methods*, *26*(6), 1481-1496.

Liu, S., Qin, Y., Xie, Z., & Zhang, J. (2020). The spatio-temporal characteristics and influencing factors of Covid-19 spread in Shenzhen, China—an analysis based on 417 cases. *International Journal of Environmental Research and Public Health*, *17*(20), 7450.

Ma, Q., Gao, J., Zhang, W., Wang, L., Li, M., Shi, J., Zhai, Y., Sun, D., Wang, L., & Chen, B. (2021). Spatio-temporal distribution characteristics of COVID-19 in China: a city-level modeling study. *BMC Infectious Diseases*, *21*, 1-14.

Mollalo, A., Rivera, K. M., & Vahabi, N. (2021). Spatial statistical analysis of pre-existing mortalities of 20 diseases with COVID-19 mortalities in the continental United States. *Sustainable cities and society*, *67*, 102738.

Moraga, P. (2023). *Spatial statistics for data science: theory and practice with R*. CRC Press.
