# Supplementary material for: Application of Spatial Analysis on Electronic Health Records to Characterize Patient Phenotypes: Systematic Review
Source: JMIR Med Inform. 2024 Oct 15;12:e56343. doi: 10.2196/56343 (PMC11522649; doi:10.2196/56343)
Supplement: Multimedia Appendix 3 [file medinform_v12i1e56343_app3.docx]

|  | Definitions of clinical phenotypes for each publication (row), including any validation methods and the primary spatial techniques employed.  *This represents a summary of the phenotype definitions. | | | |  | |
| --- | --- | --- | --- | --- | --- | --- |
| Article | | **Validation**  **(if any)** | **Phenotype Definition Summary*** | **Primary Spatial Methods** | |  |
| (Wilson et al., 2022) | |  | Age ≥18 yrs., documentation of BP (at least 1 repeat within 120 days of the first measurement) resided in census tracts with >1,000 observations. | Spatial and temporal buffers | |  |
| (Grunwell, Opolka, Mason, & Fitzpatrick, 2022) | | Chart review | Age 6 to 17 yrs., asthma (problem list) & admitted to the PICU at [facility] and [facility] between [date range] | Hot spot detection | |  |
| (S. Xie, Greenblatt, Levy, & Himes, 2017) | |  | Age ≥18 yrs., encounters that occurred between [date range] and included an asthma (ICD9) | Generalized additive model &  Density map | |  |
| (Lipner et al., 2017) | |  | Diagnosed of NTM, treated at [facility], resident in [area] during [date range] excluding cystic fibrosis | Spatial scan statistics &  Moran’s I test | |  |
| (Hanna-Attisha, LaChance, Sadler, & Champney Schnepp, 2016) | |  | Age 5 and younger with a blood lead level lab test and living within the city of Flint, Michigan. | Ordinary Kriging | |  |
| (Pearson & Werth, 2019) | |  | Age ≥18 yrs., seen at derm or rheumatology clinics at [facility], the primary residence of US ZCTA, and diagnosis of dermatomyositis noted by ICD 9 or 10 codes | Global and local Moran’s indices | |  |
| (Schwartz et al., 2011) | |  | Age 5 to <18 years with at least one usable height and weight measure; Residing in [area] | Network buffer | |  |
| (Sharif-Askary et al., 2018) | | Chart review by two independent clinical reviewers | LTFU is defined by the International Consortium for Health Outcomes Measurement Standard Set of Outcome Measures for Cleft Lip/Palate; Diagnosed and treated for cleft lip/palate excluding other conditions, residing in [area] | Spatial variogram &  generalized linear geostatistical model within a Bayesian framework | |  |
| (Carey et al., 2021) | | Chart review | Proven or probable coccidioidomycosis (published methodology of iterative querying each of 7 different types of clinical and diagnostic data: ICD9/10, laboratory, microbiology, pathology, radiology, pharmacy, and composite data) | Spatio-temporal modeling | |  |
| (S. J. Xie et al., 2023) | | eMERGE-3 phenotype | Age ≥18 yrs., primary care patients, diagnosed with depression (eMERGE-3 PheKB’s Depression Phenotype ‘2/30/180 rule’) and obesity (NIDDK definition based on recorded BMI and ICD9/10 codes) | Spatial Empirical-Bayes adjustment &  Global Moran’s I &  Local Moran’s I | |  |
| (Garg et al., 2023) | |  | Age ≥18 yrs., without hypertension (defined [list of vitals] or having ICD9s), diabetes (deﬁned any [lab values] or having ICD9s), or CKD (deﬁned by a single [lab value] or an ICD9); [date range] | Global Moran’s I &  Local Moran’s I | |  |
| (Ghazi, Drawz, & Berman, 2022) | | Chart review, random 100 charts | Age ≥18 yrs.; geocoded address from [area]; Outpatient visits, 3 or more creatinine measures (between [date range]), at least one primary care visit before the creatinine measure | Gaussian weighted kernel +  K-function test statistic | |  |
| (Chang et al., 2015) | |  | Asthma ICD9 associated with CPT codes for discharge/office visits; Controls were asthma ICD9 not associated with CPT codes for discharge/office visit, but were seen ≥ once at [facility] [date range] | Generalized additive model | |  |
| (Oyana, Podila, Wesley, Lomnicki, & Cormier, 2017) | |  | ≤18 yrs. old asthma patients identified using ICD 9-CM codes residing in the [area] | Kulldorff’s retrospective space-time statistics | |  |
| (Lê-Scherban et al., 2019) | |  | At least one visit to [facilities] in 2016, resided in [area]. Diabetes control aged 18–75 yrs, diagnosis of type 1 or 2 diabetes. Hypertension controls aged 18–85 years, diagnosis of hypertension | Getis-Ord Gi* | |  |
| (Lantos et al., 2018) | |  | Screened for multicenter trial of CMV hyperimmune globulin in pregnant women who acquired CMV infection | Generalized additive models &  standard deviation ellipse | |  |
| (Sun et al., 2022) | |  | Women who gave birth to singleton children; [list of exclusions] | Empirical Bayesian kriging &  Moran’s I | |  |
| (Davidson et al., 2018) | |  | Age ≥18 yrs., received care in either [facility] between [date range], diagnosis of depression by at least one ICD9 code, and have a valid address during the last visit | Spatial rate smoothing &  Local Indicators of Spatial  Association | |  |
| (Casey, James, Rudolph, Wu, & Schwartz, 2016) | |  | Delivered neonates (ICD9 codes) at [facilities] between [date range]; [list of exclusions] | Semivariograms &  buffer analysis | |  |
| (Lieu, Ray, Klein, Chung, & Kulldorff, 2015) | |  | Infants who received care from birth to 36 months, with >=2 primary care visits by their first birthday | Spatial scan statistics | |  |
| (Liu et al., 2021) | |  | Pediatric who underwent scheduled surgeries; living in [area]; two [departments]; [date range] | Global Moran I &  empirical Bayesian estimation &  Spatial Durbin model | |  |
| (Siegel et al., 2022) | |  | TNBC from the genomics database and “not-TNBC” cases diagnosed between [date range]; EHR data were on obesity and alcohol use disorder | Kernel Density Estimation | |  |
| (Immergluck et al., 2019) | | CDC's 'community-associated' definition | Children treated for CA-MRSA (ICD 9-CM); first positive culture during a single hospitalization within or at 48 hours of admission; and resided within [area] | Spatially adaptive filter | |  |
| (Tabano, Bol, Newcomer, Barrow, & Daley, 2017) | |  | Age ≥18 yrs.; obesity (BMI ≥ 30 kg m2); Plausible weight and height limits; Pregnant (ICD9 code OR procedure code for pregnancy or delivery) were excluded; [date range] | Moran’s I statistic &  Empirical Bayes Estimates | |  |
| (Mayne, Pellissier, & Kershaw, 2019) | |  | Singleton births at [facility] between [date range]; Lived in [area]; could be geocoded successfully; did not have missing study covariates of gestational age, birth weight, and problem lists/diagnostic codes | Semivariogram model &  Ordinary kriging &  IDW | |  |
| (Ali et al., 2019) | |  | Children with CO-MRSA (ICD9CM and positive culture resistant to oxacillin within 48 hours of hospital admission or all outpatients); Controls are children with unintentional traumatic brain injury (ICD9CM) | Getis-Ord Gi* statistic &  Space Time Cube &  Emerging Hot Spot Analysis | |  |
| (Bravo, Anthopolos, Kimbro, & Miranda, 2018) | |  | Age ≥18 yrs.; valid address in [area]; records beyond just lab test results; diabetes criteria based on ICD9 codes; medication utilization; or combinations of lab results | Bayesian Intrinsic conditional autoregressive | |  |
| (Bravo, Batch, & Miranda, 2019) | |  | Age ≥18 yrs., valid address in [area]; records beyond just lab tests; Hypertension (ICD9 codes) | Spatial Bayesian models | |  |
| (Soares, Dewalle, & Marsh, 2017) | |  | All patients receiving care at the neurodevelopmental pediatric service |  | |  |
| (Cobert et al., 2020) | |  | Admitted to [facilities] with a drug-related admission or drug poisoning-related (ICD9/10) | Hierarchical Bayesian spatial models | |  |
| (Mayne, Yellayi, Pool, Grobman, & Kershaw, 2018) | |  | Non-Hispanic black women delivering singleton births with hypertensive disorder (problem lists and ICD) plus those with gestational hypertension and pre-eclampsia | Local Getis-Ord Gi* statistic | |  |
| (Bravo, Anthopolos, & Miranda, 2019) | | eMERGE, SUPREME-DM, DDC | Age ≥18 yrs. at most recent visit and residing within [area] and with T2DM (ICD9, medications, labs) | Spatial Bayesian models | |  |
| (Kane et al., 2023) | |  | All proband participants—affected children—enrolled between [date range] | Point density | |  |
| (Georgantopoulos et al., 2020) | |  | Prostate Cancer diagnosed and treated at a VA facility residing in SC and identified by primary PrCA diagnosis in the cancer registry; non-cancer controls from SC; [date range] | Bayesian multivariate conditional autoregressive model &  Global Moran’s I | |  |
| (Jilcott et al., 2011) | |  | Age 8 to <18 yrs., address in [area] zip code, receiving well child check-ups [facility] during [date range] | GIS-based network analysis (Service Area) | |  |
| (Brooks, Brown, Liu, & Siegel, 2020) | |  | Tested for COVID-19 between [date range] | Spatial Scan Statistics | |  |
| (Zhan et al., 2021) | |  | >49 yrs. old, CRC screening (stool test within the last year or a colonoscopy within the previous 10 yrs.) | Spatial Scan Statistics | |  |
| (Patterson & Grossman, 2017) | |  | Non-Subdivided ICD9 codes from 001 to 799; Counties in the continental United States and to the ICD-9 codes that have data for two-thirds or more of the counties | Kriging | |  |
| (DeMass, Gupta, Self, Thomas, & Rudisill, 2023) | |  | Aged ≥18 yrs., engaged in ambulatory care and condition management, inpatient case management, or community health in South Carolina’s central (Midlands) and northwestern (Upstate) regions | Bayesian negative binomial hurdle models | |  |
| (Sidell et al., 2022) | |  | Diagnosed with COVID-19 by PCR result or ICD10/internal diagnosis codes; Excluding those who had negative results within two weeks following a positive result; [date range] | Moran's I &  Generalized linear mixed effects models and spatial autocorrelation | |  |
| (Gaudio et al., 2023) | |  | Radiation treatment interruptions defined as ≥5 (major) and ≥2 to 4 (minor) unplanned cancellations | Local bivariate Moran’s | |  |
| (Winckler et al., 2023) | |  | Age 5 to <18 yrs., valid home address, an ED or hospital admission for a primary reason of known mental health diagnosis or associated mental health symptom | Anselin Local Morans’ I | |  |
| (Beck, Riley, Taylor, Brokamp, & Kahn, 2018) | |  | <18 yrs. old, hospitalizations at [facility] from [area] | Point density | |  |
| (Kersten et al., 2018) | |  | Children 1 day to 18 years old in [area], visited an ED and/or adjoined urgent care department at any of 6 hospital sites within 3 medical systems between [date range] | Getis-Ord Gi* statistic | |  |
| (Zhao, Norton, & Hanrahan, 2021) | |  | Aged 5–17 yrs. as of their most recent BMI data during the enrollment calendar year | Bayesian small area estimation | |  |
| (Samuels et al., 2022) | |  | Living in [area] who were seen for an asthma-related problem (ICD9/10) in the ED [facility] | Spatial join | |  |
| (Lantos et al., 2017) | |  | Pregnant women enrolled in the clinical trial of CMV hyperimmunoglobulin to prevent perinatal transmission of or congenital CMV infection | Spatial generalized additive models &  2-standard deviation ellipse | |  |
| (Epstein et al., 2014) | |  | Children admitted to the PICU at [facility] with a primary residence in [area] | Moran’s I | |  |
| (Wakefield et al., 2020) | |  | Treated consecutively with external beam radiation therapy | Spatial Overlay | |  |

Ali, F., Immergluck, L. C., Leong, T., Waller, L., Malhotra, K., Jerris, R. C., . . . Rust, G. S. (2019). A Spatial Analysis of Health Disparities Associated with Antibiotic Resistant Infections in Children Living in Atlanta (2002-2010). *EGEMS (Wash DC), 7*(1), 50. doi:10.5334/egems.308

Beck, A. F., Riley, C. L., Taylor, S. C., Brokamp, C., & Kahn, R. S. (2018). Pervasive Income-Based Disparities In Inpatient Bed-Day Rates Across Conditions And Subspecialties. *Health Aff (Millwood), 37*(4), 551-559. doi:10.1377/hlthaff.2017.1280

Bravo, M. A., Anthopolos, R., Kimbro, R. T., & Miranda, M. L. (2018). Residential Racial Isolation and Spatial Patterning of Type 2 Diabetes Mellitus in Durham, North Carolina. *Am J Epidemiol, 187*(7), 1467-1476. doi:10.1093/aje/kwy026

Bravo, M. A., Anthopolos, R., & Miranda, M. L. (2019). Characteristics of the built environment and spatial patterning of type 2 diabetes in the urban core of Durham, North Carolina. *J Epidemiol Community Health, 73*(4), 303-310. doi:10.1136/jech-2018-211064

Bravo, M. A., Batch, B. C., & Miranda, M. L. (2019). Residential Racial Isolation and Spatial Patterning of Hypertension in Durham, North Carolina. *Prev Chronic Dis, 16*, E36. doi:10.5888/pcd16.180445

Brooks, M., Brown, C., Liu, W., & Siegel, S. D. (2020). Mapping the ChristianaCare response to COVID-19:: Clinical insights from the Value Institute's Geospatial Analytics Core. *Dela J Public Health, 6*(2), 66-70. doi:10.32481/djph.2020.07.018

Carey, A., Gorris, M. E., Chiller, T., Jackson, B., Beadles, W., & Webb, B. J. (2021). Epidemiology, clinical features, and outcomes of coccidioidomycosis, Utah, 2006–2015. *Emerging Infectious Diseases, 27*(9), 2269.

Casey, J. A., James, P., Rudolph, K. E., Wu, C. D., & Schwartz, B. S. (2016). Greenness and Birth Outcomes in a Range of Pennsylvania Communities. *Int J Environ Res Public Health, 13*(3). doi:10.3390/ijerph13030311

Chang, T. S., Gangnon, R. E., David Page, C., Buckingham, W. R., Tandias, A., Cowan, K. J., . . . Guilbert, T. W. (2015). Sparse modeling of spatial environmental variables associated with asthma. *J Biomed Inform, 53*, 320-329. doi:10.1016/j.jbi.2014.12.005

Cobert, J., Lantos, P. M., Janko, M. M., Williams, D. G. A., Raghunathan, K., Krishnamoorthy, V., . . . Gulur, P. (2020). Geospatial Variations and Neighborhood Deprivation in Drug-Related Admissions and Overdoses. *J Urban Health, 97*(6), 814-822. doi:10.1007/s11524-020-00436-8

Davidson, A. J., Xu, S., Oronce, C. I. A., Durfee, M. J., McCormick, E. V., Steiner, J. F., . . . Beck, A. (2018). Monitoring Depression Rates in an Urban Community: Use of Electronic Health Records. *J Public Health Manag Pract, 24*(6), E6-e14. doi:10.1097/phh.0000000000000751

DeMass, R., Gupta, D., Self, S., Thomas, D., & Rudisill, C. (2023). Emergency department use and geospatial variation in social determinants of health: a pilot study from South Carolina. *BMC Public Health, 23*(1), 1527. doi:10.1186/s12889-023-16136-2

Epstein, D., Reibel, M., Unger, J. B., Cockburn, M., Escobedo, L. A., Kale, D. C., . . . Gold, J. I. (2014). The effect of neighborhood and individual characteristics on pediatric critical illness. *Journal of community health, 39*, 753-759.

Garg, G., Tedla, Y. G., Ghosh, A. S., Mohottige, D., Kolak, M., Wolf, M., & Kho, A. (2023). Supermarket Proximity and Risk of Hypertension, Diabetes, and CKD: A Retrospective Cohort Study. *Am J Kidney Dis, 81*(2), 168-178. doi:10.1053/j.ajkd.2022.07.008

Gaudio, E., Ammar, N., Gunturkun, F., Akkus, C., Brakefield, W., Wakefield, D. V., . . . Schwartz, D. L. (2023). Defining Radiation Treatment Interruption Rates During the COVID-19 Pandemic: Findings From an Academic Center in an Underserved Urban Setting. *Int J Radiat Oncol Biol Phys, 116*(2), 379-393. doi:10.1016/j.ijrobp.2022.09.073

Georgantopoulos, P., Eberth, J. M., Cai, B., Emrich, C., Rao, G., Bennett, C. L., . . . Hébert, J. R. (2020). Patient- and area-level predictors of prostate cancer among South Carolina veterans: a spatial analysis. *Cancer Causes Control, 31*(3), 209-220. doi:10.1007/s10552-019-01263-2

Ghazi, L., Drawz, P. E., & Berman, J. D. (2022). The association between fine particulate matter (PM(2.5)) and chronic kidney disease using electronic health record data in urban Minnesota. *J Expo Sci Environ Epidemiol, 32*(4), 583-589. doi:10.1038/s41370-021-00351-3

Grunwell, J. R., Opolka, C., Mason, C., & Fitzpatrick, A. M. (2022). Geospatial Analysis of Social Determinants of Health Identifies Neighborhood Hot Spots Associated With Pediatric Intensive Care Use for Life-Threatening Asthma. *J Allergy Clin Immunol Pract, 10*(4), 981-991.e981. doi:10.1016/j.jaip.2021.10.065

Hanna-Attisha, M., LaChance, J., Sadler, R. C., & Champney Schnepp, A. (2016). Elevated Blood Lead Levels in Children Associated With the Flint Drinking Water Crisis: A Spatial Analysis of Risk and Public Health Response. *Am J Public Health, 106*(2), 283-290. doi:10.2105/ajph.2015.303003

Immergluck, L. C., Leong, T., Malhotra, K., Parker, T. C., Ali, F., Jerris, R. C., & Rust, G. S. (2019). Geographic surveillance of community associated MRSA infections in children using electronic health record data. *BMC Infect Dis, 19*(1), 170. doi:10.1186/s12879-019-3682-3

Jilcott, S. B., Wade, S., McGuirt, J. T., Wu, Q., Lazorick, S., & Moore, J. B. (2011). The association between the food environment and weight status among eastern North Carolina youth. *Public Health Nutr, 14*(9), 1610-1617. doi:10.1017/s1368980011000668

Kane, N. J., Cohen, A. S., Berrios, C., Jones, B., Pastinen, T., & Hoffman, M. A. (2023). Committing to genomic answers for all kids: Evaluating inequity in genomic research enrollment. *Genetics in Medicine, 25*(9), 100895.

Kersten, E. E., Adler, N. E., Gottlieb, L., Jutte, D. P., Robinson, S., Roundfield, K., & LeWinn, K. Z. (2018). Neighborhood Child Opportunity and Individual-Level Pediatric Acute Care Use and Diagnoses. *Pediatrics, 141*(5). doi:10.1542/peds.2017-2309

Lantos, P. M., Hoffman, K., Permar, S. R., Jackson, P., Hughes, B. L., Kind, A., & Swamy, G. (2018). Neighborhood Disadvantage is Associated with High Cytomegalovirus Seroprevalence in Pregnancy. *J Racial Ethn Health Disparities, 5*(4), 782-786. doi:10.1007/s40615-017-0423-4

Lantos, P. M., Hoffman, K., Permar, S. R., Jackson, P., Hughes, B. L., & Swamy, G. K. (2017). Geographic Disparities in Cytomegalovirus Infection During Pregnancy. *J Pediatric Infect Dis Soc, 6*(3), e55-e61. doi:10.1093/jpids/piw088

Lê-Scherban, F., Ballester, L., Castro, J. C., Cohen, S., Melly, S., Moore, K., & Buehler, J. W. (2019). Identifying neighborhood characteristics associated with diabetes and hypertension control in an urban African-American population using geo-linked electronic health records. *Prev Med Rep, 15*, 100953. doi:10.1016/j.pmedr.2019.100953

Lieu, T. A., Ray, G. T., Klein, N. P., Chung, C., & Kulldorff, M. (2015). Geographic clusters in underimmunization and vaccine refusal. *Pediatrics, 135*(2), 280-289. doi:10.1542/peds.2014-2715

Lipner, E. M., Knox, D., French, J., Rudman, J., Strong, M., & Crooks, J. L. (2017). A Geospatial Epidemiologic Analysis of Nontuberculous Mycobacterial Infection: An Ecological Study in Colorado. *Ann Am Thorac Soc, 14*(10), 1523-1532. doi:10.1513/AnnalsATS.201701-081OC

Liu, L., Ni, Y., Beck, A. F., Brokamp, C., Ramphul, R. C., Highfield, L. D., . . . Pratap, J. N. (2021). Understanding Pediatric Surgery Cancellation: Geospatial Analysis. *J Med Internet Res, 23*(9), e26231. doi:10.2196/26231

Mayne, S. L., Pellissier, B. F., & Kershaw, K. N. (2019). Neighborhood Physical Disorder and Adverse Pregnancy Outcomes among Women in Chicago: a Cross-Sectional Analysis of Electronic Health Record Data. *J Urban Health, 96*(6), 823-834. doi:10.1007/s11524-019-00401-0

Mayne, S. L., Yellayi, D., Pool, L. R., Grobman, W. A., & Kershaw, K. N. (2018). Racial Residential Segregation and Hypertensive Disorder of Pregnancy Among Women in Chicago: Analysis of Electronic Health Record Data. *Am J Hypertens, 31*(11), 1221-1227. doi:10.1093/ajh/hpy112

Oyana, T. J., Podila, P., Wesley, J. M., Lomnicki, S., & Cormier, S. (2017). Spatiotemporal patterns of childhood asthma hospitalization and utilization in Memphis Metropolitan Area from 2005 to 2015. *J Asthma, 54*(8), 842-855. doi:10.1080/02770903.2016.1277537

Patterson, M. T., & Grossman, R. L. (2017). Detecting Spatial Patterns of Disease in Large Collections of Electronic Medical Records Using Neighbor-Based Bootstrapping. *Big Data, 5*(3), 213-224. doi:10.1089/big.2017.0028

Pearson, D. R., & Werth, V. P. (2019). Geospatial Correlation of Amyopathic Dermatomyositis With Fixed Sources of Airborne Pollution: A Retrospective Cohort Study. *Front Med (Lausanne), 6*, 85. doi:10.3389/fmed.2019.00085

Samuels, E. A., Taylor, R. A., Pendyal, A., Shojaee, A., Mainardi, A. S., Lemire, E. R., . . . Haber, A. L. (2022). Mapping emergency department asthma visits to identify poor-quality housing in New Haven, CT, USA: a retrospective cohort study. *The Lancet Public Health, 7*(8), e694-e704.

Schwartz, B. S., Stewart, W. F., Godby, S., Pollak, J., Dewalle, J., Larson, S., . . . Glass, T. A. (2011). Body mass index and the built and social environments in children and adolescents using electronic health records. *Am J Prev Med, 41*(4), e17-28. doi:10.1016/j.amepre.2011.06.038

Sharif-Askary, B., Bittar, P. G., Farjat, A. E., Liu, B., Vissoci, J. R. N., & Allori, A. C. (2018). Geospatial Analysis of Risk Factors Contributing to Loss to Follow-up in Cleft Lip/Palate Care. *Plast Reconstr Surg Glob Open, 6*(9), e1910. doi:10.1097/gox.0000000000001910

Sidell, M. A., Chen, Z., Huang, B. Z., Chow, T., Eckel, S. P., Martinez, M. P., . . . Xiang, A. H. (2022). Ambient air pollution and COVID-19 incidence during four 2020-2021 case surges. *Environ Res, 208*, 112758. doi:10.1016/j.envres.2022.112758

Siegel, S. D., Brooks, M. M., Sims-Mourtada, J., Schug, Z. T., Leonard, D. J., Petrelli, N., & Curriero, F. C. (2022). A Population Health Assessment in a Community Cancer Center Catchment Area: Triple-Negative Breast Cancer, Alcohol Use, and Obesity in New Castle County, Delaware. *Cancer Epidemiol Biomarkers Prev, 31*(1), 108-116. doi:10.1158/1055-9965.Epi-21-1031

Soares, N., Dewalle, J., & Marsh, B. (2017). Utilizing patient geographic information system data to plan telemedicine service locations. *J Am Med Inform Assoc, 24*(5), 891-896. doi:10.1093/jamia/ocx011

Sun, Y., Li, X., Benmarhnia, T., Chen, J. C., Avila, C., Sacks, D. A., . . . Wu, J. (2022). Exposure to air pollutant mixture and gestational diabetes mellitus in Southern California: Results from electronic health record data of a large pregnancy cohort. *Environ Int, 158*, 106888. doi:10.1016/j.envint.2021.106888

Tabano, D. C., Bol, K., Newcomer, S. R., Barrow, J. C., & Daley, M. F. (2017). The Spatial Distribution of Adult Obesity Prevalence in Denver County, Colorado: An Empirical Bayes Approach to Adjust EHR-Derived Small Area Estimates. *EGEMS (Wash DC), 5*(1), 24. doi:10.5334/egems.245

Wakefield, D. V., Carnell, M., Dove, A. P. H., Edmonston, D. Y., Garner, W. B., Hubler, A., . . . Schwartz, D. L. (2020). Location as Destiny: Identifying Geospatial Disparities in Radiation Treatment Interruption by Neighborhood, Race, and Insurance. *Int J Radiat Oncol Biol Phys, 107*(4), 815-826. doi:10.1016/j.ijrobp.2020.03.016

Wilson, W. W., Chua, R. F. M., Wei, P., Besser, S. A., Tung, E. L., Kolak, M., & Tabit, C. E. (2022). Association Between Acute Exposure to Crime and Individual Systolic Blood Pressure. *Am J Prev Med, 62*(1), 87-94. doi:10.1016/j.amepre.2021.06.017

Winckler, B., Nguyen, M., Khare, M., Patel, A., Crandal, B., Jenkins, W., . . . Rhee, K. E. (2023). Geographic Variation in Acute Pediatric Mental Health Utilization. *Acad Pediatr, 23*(2), 448-456. doi:10.1016/j.acap.2022.07.026

Xie, S., Greenblatt, R., Levy, M. Z., & Himes, B. E. (2017). Enhancing Electronic Health Record Data with Geospatial Information. *AMIA Jt Summits Transl Sci Proc, 2017*, 123-132.

Xie, S. J., Kapos, F. P., Mooney, S. J., Mooney, S., Stephens, K. A., Chen, C., . . . Pratap, A. (2023). Geospatial divide in real-world EHR data: Analytical workflow to assess regional biases and potential impact on health equity. *AMIA Jt Summits Transl Sci Proc, 2023*, 572-581.

Zhan, F. B., Morshed, N., Kluz, N., Candelaria, B., Baykal-Caglar, E., Khurshid, A., & Pignone, M. P. (2021). Spatial Insights for Understanding Colorectal Cancer Screening in Disproportionately Affected Populations, Central Texas, 2019. *Prev Chronic Dis, 18*, E20. doi:10.5888/pcd18.200362

Zhao, Y.-Q., Norton, D., & Hanrahan, L. (2021). Small area estimation and childhood obesity surveillance using electronic health records. *PLoS One, 16*(2), e0247476.
